# Expression Profile and Clinical Significance of MicroRNAs in Papillary Thyroid Carcinoma

**DOI:** 10.3390/molecules190811586

**Published:** 2014-08-05

**Authors:** You Peng, Chen Li, Ding-Cun Luo, Jin-Wang Ding, Wo Zhang, Gang Pan

**Affiliations:** 1 Department of Oncological Surgery, Hangzhou First People’s Hospital, Hangzhou 310006, China; E-Mails: doctor3663@163.com (Y.P.); zjlsdjw@163.com (J.-W.D.); zwwyntx@sina.com (W.Z.); xjxsh@163.com (G.P.); 2 Department of General Surgery, Wuxi Second People’s Hospital, Wuxi 214000, China; E-Mail: bullfrog007@sina.com; 3 Department of Oncological Surgery, Wushan District of Hangzhou First People’s Hospital, Hangzhou Cancer Hospital, Hangzhou 310002, China

**Keywords:** papillary thyroid carcinoma, miRNA, invasiveness

## Abstract

This study screened microRNAs (miRNAs) that are abnormally expressed in papillary thyroid carcinoma (PTC) tissues to identify PTC and nodular goiter and the degree of PTC malignancy. A total of 51 thyroid tumor tissue specimens paired with adjacent normal thyroid tissues were obtained from the Department of Surgical Oncology of Hangzhou First People’s Hospital from June-December 2011. miRNA expression profiles were examined by microarrays and validated by quantitative real-time PCR (qRT-PCR). Expression levels of the miRNAs were analyzed to assess if they were associated with selected clinicopathological features. Eleven miRNAs were significantly differentially expressed between nodular goiter and PTC and between highly invasive and low invasive PTC. miR-199b-5p and miR-30a-3p were significantly differentially expressed among the three groups. miR-30a-3p, miR-122-5p, miR-136-5p, miR-146b-5p and miR-199b-5p were selected for further study by qRT-PCR and miR-146b-5p, miR-199b-5p and miR-30a-3p were different between the PTC and nodular goiter groups. miR-199b-5p was over-expressed in PTC patients with extrathyroidal invasion and cervical lymph node metastasis. In conclusion miR-146b-5p, miR-30a-3p, and miR-199b-5p may serve as biomarkers for the diagnosis of PTC and miR-199b-5p is associated with PTC invasiveness.

## 1. Introduction

With an incidence of 2% thyroid carcinoma is the most common form of endocrine system malignancy [1,2]. Papillary thyroid carcinoma (PTC) accounts for around 90% of these cases and this number is still increasing [1,3]. Most PTC shows low malignancy and invasiveness. The chance of recovery is good, as up to 80% of these patients can live 10 more years, but in a few cases the disease can recur, transform and cause death [4]. As there are no obvious symptoms in PTC patients and painless nodules are the only clinical symptoms, a definite diagnosis of PTC cannot be made, even with nodules, making early or timely diagnosis quite difficult [5]. Unfortunately, there were no effective methods for identifying the level of malignancy and invasiveness but this is changing with the discovery of molecular markers for PTC malignancy, for example the T1799A *BRAF* mutation in the mitogen-activated protein kinase pathway [6,7]. This marker is associated with an increased risk of tumor recurrence, lymph node metastasis, extrathyroidal extension and advanced stage thyroid cancer [7]. Consequently, new prognostic markers are currently being explored for the effective diagnosis and treatment of PTC, and research into the potential for microRNA (miRNA) use in this area is currently a popular research topic [8,9,10,11,12,13,14]. miRNAs are small non-protein coding RNAs that have been shown to have roles in a wide range of cellular processes including cancer and metastasis [15]. The roles of miRNAs in PTC are still being revealed, but it has been shown that some aberrant miRNAs are expressed more highly in PTC [16]. It has also been suggested that circulating miRNAs might be used as markers for the existence of PTC [17]. It is apparent, however, that the differential expression of miRNAs within PTC tissues compared to non-tumor tissues may serve as important measures of the invasiveness and therefore patient outcome [16]. This study sought to improve the clinical competence of identifying PTC and multinodular goiter and its invasiveness by investigating miRNAs that are specifically expressed in PTC tissues, and analyzing their correlation with clinical-pathological features. We found that miRNAs, as suggested previously, may be used as biomarkers for PTC and that there are differentially expressed miRNAs that may be used to measure invasiveness. These results present important information regarding the use of miRNAs in the diagnosis of PTC.

## 2. Results and Discussion

### 2.1. Results

#### 2.1.1. Quality Evaluation of the Total miRNAs

The miRNAs were evaluated using a UV-2401PC spectrophotometer (Shimadzu Corporation, Tokyo, Japan) and the value of OD_260 nm_/OD_280 nm_ ranged from 1.8 to 2.2, indicating that the sample was not contaminated by DNA or protein.

#### 2.1.2. Results of miRNA Microarray

Results of the miRNA microarray are shown in Table 1 and Supplementary Figure S1. Eleven miRNAs whose expression increased or decreased more than 2-fold in in PTC compared to benign nodular goiter were identified, including seven miRNAs that were up-regulated (miR-146b-5p, miR-222-3p, miR-221-3p, miR-10b-5p, miR-199a-3p/miR-199b-3p, miR-203 and miR-32-5p) and four miRNAs that were down-regulated (miR-122-5p, miR-183-3p, miR-149-3p, and miR-514a-3sp) in PTC compared to benign nodular goiter (groups A and B *vs.* C). In the comparison of invasiveness, four miRNAs were found to be differentially expressed by more than 2-fold, including two up-regulated miRNAs (miR-136-5p and miR-199a-5p) and two down-regulated miRNAs (miR-513 and miR-1243) in the high invasion compared to the low invasion group (group A *vs.* group B).

**Table 1:** miRNA microarray results showing differential expression between groups.

miRNA	A *vs.* B	A *vs.* C	B *vs.* C
hsa-miR-146b-5p	1.08	43.36	40.25
hsa-miR-136-5p	3.11	5.46	1.75
hsa-miR-199b-5p	2.05	11.48	5.61
hsa-miR-30a-3p	0.49	0.21	0.42
hsa-miR-122-5p	1.08	0.28	0.26
hsa-miR-222-3p	0.84	2.87	3.41
hsa-miR-221-3p	0.71	2.44	3.42
hsa-miR-10b-5p	0.97	15.60	16.01
hsa-miR-199a-3p/hsa-miR-199b-3p	1.95	29.27	14.99
hsa-miR-203	0.87	12.43	14.30
hsa-miR-32-5p	0.89	10.87	12.22
hsa-miR-183-3p	1.33	0.49	0.37
hsa-miR-199a-5p	2.02	3.95	1.95
hsa-miR-149-3p	1.02	0.50	0.49
hsa-miR-514a-3p	0.60	0.14	0.24
hsa-miR-1243	0.13	0.07	0.50
hsa-miR-513b	0.32	0.23	0.73

A is high invasive PTC group; B is low invasive PTC group; C is benign thyroid nodular goiter group; PTC, papillary thyroid carcinoma. The results are presented as fold changes A *vs.* B is (Fluorescence intensity of A)/(Fluorescence intensity of B), A *vs.* C is (Fluorescence intensity of A)/(Fluorescence intensity of C), B *vs.* C is (Fluorescence intensity of B)/(Fluorescence intensity of C).

Another two miRNAs, miR-199b-5p and miR-30a-3p, were observed to be differentially expressed by more than 2-fold between the three groups. It has been demonstrated that miRNA functions by inhibiting the expression of targeted genes. We screened for the target genes of the miRNAs by using DIANA-microT (Strict, Loose, beta version), PicTar and TargetScan. Seven miRNAs were predicted to be associated with PTC and they were miR-146b-5p, miR-136-5p, miR-199b-5p, miR-30a-3p, miR-122-5p, miR-222-3p and miR-221-3p. Since miR-222-3p and miR-221-3p have been thoroughly studied since their identification in the past few years [18], we chose the other five miRNAs to investigate here.

#### 2.1.3. Results of the qRT-PCR

The amplification and solubility curves showed that miR-146b-5p, miR-136-5p, miR-199b-5p, miR-30a-3p, miR-122-5p and U6 were all specifically amplified. The amplification curves were smooth, indicating that the samples were completely amplified. The solubility curves were single peaks, indicating specific amplification. The amplification curve for the empty control tube was mainly a horizontal line, indicating that the result was reliable (Supplementary Figure S2).

#### 2.1.4. Results of Real-Time RT-PCR Verification of the Microarray Analysis

The expression profiles of the miRNAs in PTC tissues, non-tumor tissues adjacent to PTC, benign thyroid nodular goiter tissues and non-nodule tissues adjacent to benign thyroid nodular goiter were compared (Table 2).

**Table 2:** Differential expression of miRNAs in different tissues

miRNAs	Tissues	Expression Levels
miR-30a-3p	T-NG	3.58 (1.75–4.27)
	T-PTC	4.66 (3.54–5.37) *
	A-NG	3.35 (2.27–3.66) ^##^
	A-PTC	2.05 (0.61–2.96) **^,##,Δ^
miR-122-5p	T-NG	10.43 (9.98–12.79)
	T-PTC	10.52 (9.20–11.96)
	A-NG	7.36 (6.66–7.98) **^,##^
	A-PTC	7.55 (6.25–8.52) **^,##^
miR-136-5p	T-NG	10.60 (9.09–12.02)
	T-PTC	10.69 (9.09–11.34)
	A-NG	7.56 (6.74–8.68) **^,##^
	A-PTC	7.76 (6.94–8.82) **^,##^
miR-146b-5p	T-NG	3.12 (2.23–4.99)
	T-PTC	−0.34 (−1.58–0.31) **
	A-NG	2.89 (1.92–4.30) ^##^
	A-PTC	2.78 (1.52–3.84) ^##^
miR-199b-5p	T-NG	7.32 (5.79–7.90)
	T-PTC	4.6s7 (3.90–5.87) **
	A-NG	1.49 (0.68–4.22) **^,##^
	A-PTC	3.31 (2.21–3.93) **^,##^

The expression values were calculated by ΔCt. * *p* < 0.05, ** *p* < 0.01 *vs.* T-NG; ^#^ *p* < 0.05, ^##^ *p* < 0.01 *vs.* T-PTC; Δ*p* < 0.05 *vs.* A-NG. T-NG: benign thyroid nodular goiter tissue (n = 15); T-PTC: papillary thyroid carcinoma tissues (*n* = 36); A-NG: non-nodular tissue adjacent to benign thyroid nodular goiter (*n* = 15); A-PTC: non-tumor tissue adjacent to PTC (*n* = 36); miRNA expression levels are presented as the median and interquartile.

The results showed that the expression of miR-30a-3p in PTC tissue was higher than in any other tissues, but its expression in non-tumor tissues adjacent to PTC was lower than in benign thyroid nodule tissue and non-nodular tissue adjacent to benign thyroid nodular goiter. miR-146b-5p was less highly expressed in PTC tissue than in other non-tumor tissues and within the non-tumor groups, its expression was not different. The expression of miR-122-5p and miR-136-5p in benign and malignant tumor tissues were higher than that in non-tumor tissues adjacent to PTC. Their expression levels were no different between benign and malignant tissue. The expression of miR-199b-5p in both benign and malignant tumor tissues was higher than in non-tumor tissues adjacent to PTC, but its expression in PTC tissue was lower than in benign thyroid nodule tissue.

To adjust for the differential expression of U6, we then analyzed the expression of miRNAs by using −∆∆CT and the results are shown in Table 3. We found that miR-30a-3p, miR-146b-5p and miR-199b-5p were differentially expressed in benign and malignant nodules. The results showed that miR-30a-3p was down-regulated and miR-146b-5p and miR-199b-5p were up-regulated, which agreed with the microarray results.

**Table 3:** Correlation analysis of miRNAs between PTC and benign thyroid nodular goiter.

miRNAs	Benign Thyroid Nodular Goiter (15 cases)	PTC (36 cases)	*p*
miR-30a-3p	−0.28 (−1.0–1.11)	−2.89 (−4.15–−1.47)	0.003
miR-122-5p	−3.66 (−4.41–−1.55)	−3.04 (−5.47–−0.99)	0.972
miR-136-5p	−2.43 (−4.51–−1.20)	−2.62 (−4.07–−1.42)	0.973
miR-146b-5p	−1.04 (−2.42–1.31)	3.72 (1.2-5.04)	0.001
miR-199b-5p	−4.68 (−7.11–−2.29)	−1.95 (−2.93–−0.04)	0.007

The expression values were calculated by (−∆∆CT). Expression levels of miRNAs are presented as the median and interquartile.

#### 2.1.5. Analysis of the Diagnostic Value of miRNAs for Distinguishing PTC from Benign Thyroid Nodular Goiter Tissues

**Figure 1:**
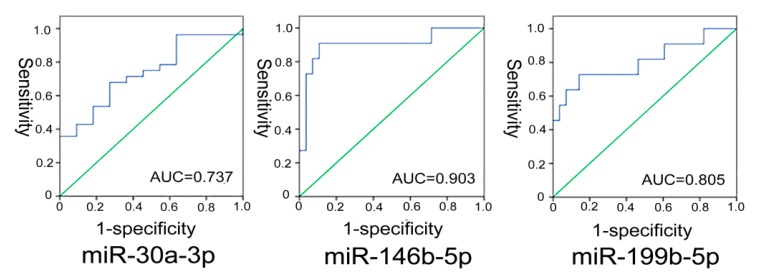
miR-30a-3p, miR-146b-5p, miR-199b-5p receiver operating characteristic (ROC) curves.

We then plotted a receiver operating characteristic (ROC) curve for miR-30a-3p, miR-146b-5p and miR-199b-5p in 51 patients; and the AUC was 0.737, 0.903 and 0.805, respectively (shown in Figure 1). The best cutoff of −∆CT for miR-30a-3p to predict PTC from benign thyroid nodular goiter tissues was −4.10 with the biggest sum of sensitivity and specificity (67.9% and 72.7%, respectively). The best cutoff of miR-146b-5p was −0.99 with sensitivity and specificity of 90.9% and 89.3%, respectively. The best cutoff of miR-199b-5p was −6.21, and sensitivity and specificity was 72.7% and 82.1%, respectively.

#### 2.1.6. Correlation Analysis between miRNAs and Clinical-Pathological Features

The association between the relative expression profiles of three miRNAs, miR-30a-3p, miR-146b-5p and miR-199b-5p, and clinical-pathological features was analyzed (Table 4). The results showed that miR-199b-5p expression was associated with extrathyroidal extension and lymph node metastasis.

**Table 4:** Correlation analysis between miRNAs and clinicopathological features.

Clinical Features	Group	n	miR-30a-3p		miR-146b-5p		miR-199b-5p	
			Expression Level	*p*	Expression Level	*p*	Expression Level	*p*
Age	≥45	21	−2.56 (−3.32–−1.27)	0.547	3.44 (0.97–3.69)	0.705	−2.54 (−3.39–−1.92)	0.319 *
	˂45	15	−1.54 (−4.39–−0.27)		1.94 (0.97–4.97)		−1.81 (−3.67–−0.13)	
Sex	Male	7	−1.73 (−3.62–0.60)	0.670	3.62 (1.12–6.12)	0.279	−0.56 (−2.84–0.17)	0.092 *
	Female	29	−2.099 (−3.69–−0.84)		2.61 (0.16–4.08)		−2.54 (−3.85–−1.33)	
Tumor size	≤1 cm	14	−1.61 (−3.88–0.33)	0.482	3.57 (1.05–4.97)	0.352	−1.63 (−2.97–0.11)	0.125 *
	˃1 cm	22	−2.329 (−3.50–−1.26)		2.51 (−1.4–4.06)		−2.67 (−5.28–−1.73)	
Number of foci	Single lesion	20	−1.97 (−3.17–−1.23)	0.058	3.84 (2.36–5.21)	0.339	−1.38 (−3.18–0.17)	0.650 *
	Multiple lesions	16	−3.60 (−4.72–−2.93)		3.47 (1.15–4.33)		−1.98 (−2.71–−1.22)	
Extrathyroidal extension	NO	27	−2.93 (−4.56–−1.56)	0.435	3.69 (1.15–5.10)	0.678	−2.51 (−2.97–−1.29)	0.047 *
	YES	9	−1.97 (−3.52–−1.47)		3.73 (3.05–3.96)		0.21 (−1.80–0.44)	
Lymph node metastasis	N_0_	15	−3.15 (−3.88–−1.56)	0.679	3.63 (1.15–4.06)	0.303	−2.54 (−3.39–−1.92)	0.010 ^#^
	N_1a_	9	−2.37 (−3.60–−1.97)		3.80 (3.47–5.45)		–2.61 (−3.65–−1.22)	
	N_1b_	12	−1.65 (−4.56–0.87)		4.97 (1.27–5.75)		0.10 (−0.62–0.29)	

The expression values were calculated by (−∆∆CT) miRNA expression levels are presented as the median and interquartile AJCC TNM staging the seventh edition [19]. * Using the Mann-Whitney U test, ^#^ Using the Kruskal-Wallis H test.

### 2.2. Discussion

#### 2.2.1. miRNA and Diagnosis of PTC

This study showed by microarray analysis that 11 miRNAs were differentially expressed, by at least 2-fold, in PTC compared to benign nodules. Seven were up-regulated (miR-146b-5p, miR-222-3p, miR-221-3p, miR-10b-5p, miR-199a-3p/miR-199b-3p, miR-203 and miR-32-5p) and four were down-regulated (miR-122-5p, miR-183-3p, miR-149-3p, and miR-514a-3p). Five of these were then verified by qRT-PCR to confirm that miR-30a-3p in PTC tissue was lower than that in any other tissues, and miR-146b-5p was more highly expressed in PTC tissue than in other non-tumor tissues. Both miR-122-5p and miR-136-5p were higher in benign and malignant tumor tissues than in adjacent tissues. miR-199b-5p was lower in both benign and malignant tumor tissues than in non-tumor tissues adjacent to PTC but its expression in PTC tissue was higher than that in benign thyroid nodule tissue. This is the first time that these expression patterns have been shown in relation to PTC. However, recently quite a few experiments have validated the correlation between miRNA expression levels and PTC. He and others found that miR-146, miR-155, miR-181, miR-221, and miR-222 showed elevated expression in PTC tissues with the help of miRNA mircroarrays [18]. Pallante and others also arrived at a similar conclusion and they proposed that the over-expression of miRNA could induce cancer in thyroid cells [20]. More recently other research has given a strong indication that miR-146, miR-221, and miR-222 are all expressed more highly in more aggressive forms of PTC and that these could be used as biomarkers for the recurrence of the disease [21,22,23,24]. Currently few studies have shown an obvious correlation between miRNA expression profiling and the subtypes of PTC histology despite the fact that PTC has many different subtypes including typical, Hurthle cell, tall cell, and the follicular variant of PTC [25].

This is, to our knowledge, the first study to show an up-regulation of miR199b-5p in PTC where previously this miRNA showed down-regulation in follicular thyroid cancer in comparison to adjacent tissue [14,26]. miR199b-5p was highest in the adjacent tissue to the PTC but clearly up-regulated in PTC in comparison to benign nodules. We are also, to our knowledge, the first to show down-regulation of miR-30a-3p and increased miR-136-5p and miR-122-5p in both the benign and cancerous nodules compared to the adjacent tissue. In this study, adjacent tissue was investigated to provide more evidence that the alterations in the miRNAs were the result of the alterations in the cancerous cells, or at least, a response to the alterations in the cancerous cells, rather than a variation in the individual patient. These expression patterns show that there may be a complex process involved with some of the miRNAs and that expression in the adjacent tissue may be influenced by cancerous and benign nodules and so should probably be considered when the miRNAs are investigated.

The major reason behind the analysis of the different expression patterns of miRNA in PTC is to investigate the application of fine needle aspiration (FNA) in clinical diagnosis. At present, FNA cannot obtain a large amount of tissue, and sometimes the accuracy using cytomorphology to diagnose benign and malignant thyroid tumors is not satisfactory. Although this is more of a problem in the follicular variant of PTC than typical PTC where diagnosis is often more straightforward. It is also difficult to diagnose the level of malignancy, and even more difficult to detect invasiveness to allow timely medical treatment. With the miRNA technique, only a few FNA samples are needed for detection. Nikiforova and some others carried out diagnostic tests with seven overexpressed miRNAs [27], they found that when the level of miRNA is doubled or more, the accuracy of tumor detection is 95%, with sensitivity of 100%, and specificity of 94%. When multiple-miRNAs are doubled or more, the accuracy of tumor detection is 98%, with sensitivity of 88%, and specificity of 94%. Our research shows that miR-30a-3p, miR-146b-5p and miR-199b-5p express differently in benign and malignant thyroid tumor tissues, and the AUC curve (the area under ROC curve) was 0.737, 0.903 and 0.805 respectively (for details, see Figure 1), therefore we conclude that miRNA has diagnostic value.

As well as the miRNAs identified here, other studies have found alternative miRNAs to be differentially expressed in PTC such as miR-335 [28], miR-135b [24], miR-206, miR-299-3p, miR-101 and miR-103 [29]. Despite the fact that the conclusions are not unanimous between all of the studies, and that there are no publicly accepted miRNA indicators, there is no denying that these results have demonstrated the potential value and advantage of miRNA techniques for PTC diagnosis.

#### 2.2.2. miRNA is Related to the Invasiveness of PTC

PTC can be classified as a highly differentiated and poorly invasive tumor, and patients recover well, with 90% likely to live ten more years [3]. But during clinical work, we also find that when compared with the majority of PTC, some cases have very different biological behavior. These cases have a large number of transformed lymph nodes, which invade tissues like the peripheral nerve, blood vessels and even muscle in the worst cases. Therefore, some scholars have investigated the correlation between miRNA and PTC [21,22,23,24,25,30]. Chou and others find that there is a significant correlation between the expression profiling of miR-222 and miR-146b [31] and extrathyroidal extension [18], and they point out that miR-146b is highly elevated in the V600E *BRAF* mutation [31]. Some others also find KIT gene expression is inhibited by the interaction between miR-146b and KIT mRNA [32]. As a receptor tyrosine kinase, KIT plays an important role in cell differentiation and proliferation. In many tumors, it acts as an anti-oncogene. Some research indicates that KIT expression is significantly lowered in PTC cell lines, so we can conclude that the high expression of miR-146 contributes to the formation of PTC. In breast cancer [33] and glioma [34], miR-146b can inhibit the expression of epidermal growth factor receptor (EGFR) in order to suppress tumor spreading, which may be one explanation for PTC showing little distant metastasis and a good prognosis.

In this research, the result of the miRNA microarray analysis indicates that six miRNAs were differentially expressed according to PTC invasiveness. miR-136-5p and miR-199a-5p were

up-regulated in highly invasive compared to low invasive PTC and miR-513 and miR-1243 were down-regulated. Importantly miR-30a-3p was down-regulated across the three groups from benign tumors to the highly invasive PTC samples and the inverse was observed with miR-199b-5p. miR-199b-5p also correlated with PTC invasiveness in the qRT-PCR result, but in qRT-PCR miR-30a-3p showed no correlation with PTC invasiveness, this difference may be a statistical error caused by relatively small samples, and requires further investigation. In previous research some scholars claim that miR-30a-3p exerts an influence on epithelial-mesenchymal transitions (EMT) through its regulation of vimentin transcription, so that tumor cells have increased invasiveness and distant metastasis capability [35]. In studying miR-1996b-5p, we find that it is highly expressed among patients with extrathyroidal invasion and cervical lymph node metastasis and this shows statistical significance (*p* = 0.047, 0.01). Meanwhile, miR-1996b-5p is involved with the occurrence and development of liver cancer [36], leukemia [37] and lung cancer [38]. According to this, we maintain that there is a positive correlation between miR-199b-5p and PTC invasiveness, so this miRNA could be helpful in evaluating PTC invasiveness and be regarded as a reference for setting up a scientific treatment schedule.

## 3. Experimental Section

### 3.1. Source of Specimens

Fifty-one thyroid samples were obtained from surgical operations conducted in the Department of Surgical Oncology of Hangzhou First People’s Hospital from January to August in 2012. None of the patients recruited in the present study received radiotherapy, chemotherapy or any other treatment before operation. 36 of the 51 cases were classical/typical PTC and 15 were benign nodular goiter diagnosed by pathology, including 13 male and 38 female patients with a median age of 48 years (range, 34–60 years). Of the 36 PTC patients included in this study 15 patients were aged less than 45 and 29 were female. The tumor size was larger than 1 cm in 22 patients and 16 had multiple lesions. Extrathyroidal extension was seen in nine patients and lymph node metastasis was in one lymph node in nine patients and 2–3 regional lymph nodes in 12 patients. There was no recurrence or distant metastasis. Detailed information of the patients has been summarized in the Table 4. After excision of the thyroid samples from the patients, both non-tumor tissues adjacent to PTC and non-nodular tissues adjacent to benign nodular goiter tissues were also taken from the above samples farthest from the focal area and preserved in liquid-nitrogen. The adjacent tissues acted as an internal control to evaluate whether the altered expression of the miRNAs was due to the cancerous cells or natural variation within the individual. They were transferred to a −80 °C freezer for long-time preservation. According to the clinical-pathological features, the samples were further divided into three groups: (1) Group A the high invasive PTC group: PTC from patients with lateral lymph node metastasis as verified by pathological features (*n* = 12). These patients are classified as N_1b_ in Table 4; (2) Group B the low invasive PTC group: PTC tissues from patients without lymph node metastasis as verified by pathological features (*n* = 15). These patients are classified as N_0_ in Table 4; (3) Group C the benign nodular goiter group: tissue from patients with nodular goiter as verified by pathological features (*n* = 15). All 51 tissue samples were used for qRT-PCR verification, and 12 of the samples were used for miRNA microarrays analysis with four samples in each of the three groups.

### 3.2. Experiment Procedure

#### 3.2.1. Microarrays Analysis of miRNA

The chip used here was a miRCURY™ LNA chip (v.16.0), tested by the Shanghai Kangcheng Biological Company (Shanghai, China). After the selection of four cases in the three sample groups (Groups A, B and C as above), the total amount of RNA was extracted with Trizol (Invitrogen, Carlsbad, CA, USA), and then miRNAs were extracted with the miRNeasy mini kit (QIAGEN) according to the manufacturers’ instructions. After quantification using a NanoDrop 1000, the total miRNA was divided into three mixed samples, and labeled using the miRCURY™ Hy3™/Hy5™ Power labeling kit (Shanghai Kangcheng Biological Company). Then the miRNA was hybridized onto the miRCURY™ LNA chip (v.16.0). After washing the slides were scanned with an Axon GenePix 4000B microarray scanner and analyzed by GenePix Pro 6.0 software (Axon, Union, CA, USA) in order to obtain values of differentially expressed miRNA in comparison to the nodular goiter miRNA sample and find those that showed a 2 fold or greater change in expression. We screened for the target genes of the differentially expressed miRNAs with DIANA-microT (Strict, Loose, beta version), PicTar and TargetScan [39]. Seven miRNAs were predicted to be associated with PTC. The predictions were based upon computer algorithm analysis. DIANA-microT searches for a positive and a negative set of miRNA recognition elements located in both the 3'-UTR and CDS regions.

#### 3.2.2. Quantitative Real-Time RT-PCR Detection of miRNAs

Because miR-222-3p and miR-221-3p have already been thoroughly studied since their first investigation [18], we chose the other five miRNAs to investigate in the present study.

#### 3.2.2.1. Design of Forward Primers

This experiment used miRbase [40] as a database to design the primers. The sequences of the forward primers are shown in Table 5.

**Table 5:** Forward primer sequences for PCR of miRNAs.

miRNAs	PCR Forward Primer
hsa-miR-146b-5p	5'-TGAGAACTGAATTCCATAGGCT-3'
hsa-miR-199b-5p	5'-CCCAGTGTTTAGACTATCTGTTC-3'
hsa-miR-30a-3p	5'-CTTTCAGTCGGATGTTTGCAGC-3'
hsa-miR-122-5p	5'-TGGAGTGTGACAATGGTGTTTG-3'
hsa-miR-136-5p	5'-ACTCCATTTGTTTTGATGATGGA-3'
U6 RNA	5'-TGCGGGTGCTCGCTTCGGCAGC-3'

Common Reverse primer used in this experiment is Uni-miR qRCR (TaKaRa Code: D352).

#### 3.2.2.2. Extraction of Total RNA

Tissue samples stored at −80 °C were placed in a mortar, with liquid nitrogen, and the tissues were rapidly ground. Total RNA was extracted using RNAiso Plus (TaKaRa Code: D9108A), and a UV-2401PC spectrophotometer (Shimadzu Corporation, Tokyo, Japan) was used to measure the OD_260 nm_ and OD_280 nm_ values of the samples to evaluate the purity of RNA based on the ratio of OD_260 nm_ to OD_280 nm_.

#### 3.2.2.3. Reverse Transcription

This study employed poly(A) to conduct the reverse transcription reaction with the One Step PrimeScript^®^ miRNA cDNA Synthesis Kit (Code: D350A, TaKaRa, Kyoto, Japan ) according to the instructions. Each reverse transcription required 1μg RNA. The composition of the reaction was 2× miRNA Reaction Buffer Mix 10 μL; 0.1% BSA 2 μL; miRNA PrimeScript^®^ RT Enzyme Mix 2 μL; made up to 20 μL with RNase free ddH_2_O. The reaction conditions were 50 °C for 60 min then 85 °C for 5 s.

#### 3.2.2.4. Amplification of PCR

Amplification of the cDNA template obtained from the above procedure was performed by PCR. The composition of the 20 μL reaction was as follows: SYBR^®^ Premix Ex TaqTM II(2×) 10 μL; PCR Forward Primer (10 μM) 0.8 μL; Uni-miR qPCR Primer (10 μM) 0.8 μL; ROX Reference Dye II(50×) 0.4 μL; cDNA solution 2 μL; ddH_2_O 6 μL. The reaction solution was compiled on ice. Each sample had two reactions, and each set of samples has a blank control. Trigger qRT-PCR reaction with ABI 7500 Real-Time System. All reactions involved initial denaturation at 95 °C for 30 s followed by 40 cycles of 95 °C for 5 s, 60 °C for 34 s. Once the reaction was finished, threshold cycles (CT) of all the reaction tubes were calculated automatically by computer. If the CT difference exceeded one, then samples were repeated. We employed (−ΔΔCT) to calculate the relative expression of the target genes. The internal reference was U6 and water was the negative control in place of the cDNA.


ΔCT = CT_(miRNA)_ − CT_(U6 RNA)_ (1)

ΔΔCT = ΔCt (T) − ΔCT (N)
(2)

T/N = 2^−ΔΔCT^ (3)

The T and N values have now been defined as T: PTC tissues or benign thyroid nodular goiter tissues; N: Non-tumor tissues adjacent to PTC or non-nodular tissues adjacent to benign thyroid nodular goiter.

### 3.3. Statistical Analysis

The software package SPSS17.0 (SPSS Inc., Chicago, IL, USA) was applied to analyze data. The Kolmogorov-Smirnov (K-S) test to test whether the data obtained was in line with normal distribution. If the data had a normal distribution, it was presented as mean ± standard deviation (SD), and analyzed by one-way analysis of variance (ANOVA) or independent sample *t* test. If the data distribution was not normal, it was presented as median (Q25, Q75), and analyzed by Mann-Whitney U test for comparison between groups, and Kruskal-Wallis H test for comparison among three or more groups. The diagnostic value of miRNA for distinguishing PTC from benign thyroid nodule tissues was analyzed with ROC curve analysis. The area under the curve (AUC) and the corresponding values of sensitivity and specificity were used to extrapolate a cut-off value. *p* < 0.05 was regarded as statistical significance.

## 4. Conclusions

We found that miR-146b-5p, miR-30a-3p, and miR-199b-5p may serve as biomarkers for the diagnosis of PTC. miR-199b-5p is associated with the invasiveness of PTC.

## Supplementary Materials

Supplementary materials can be accessed at: http://www.mdpi.com/1420-3049/19/8/11586/s1.

## Supplementary Files

Supplementary File 1
