# Supplementary material for: Expression Profile and Clinical Significance of MicroRNAs in Papillary Thyroid Carcinoma
Source: Molecules. 2014 Aug 5;19(8):11586–99. doi: 10.3390/molecules190811586 (PMC6271659; doi:10.3390/molecules190811586)
Supplement: Supplementary File 1 [file molecules-19-11586-s001.pdf]

Supplementary Information

Figure S1. Results of the miRNA microarray.

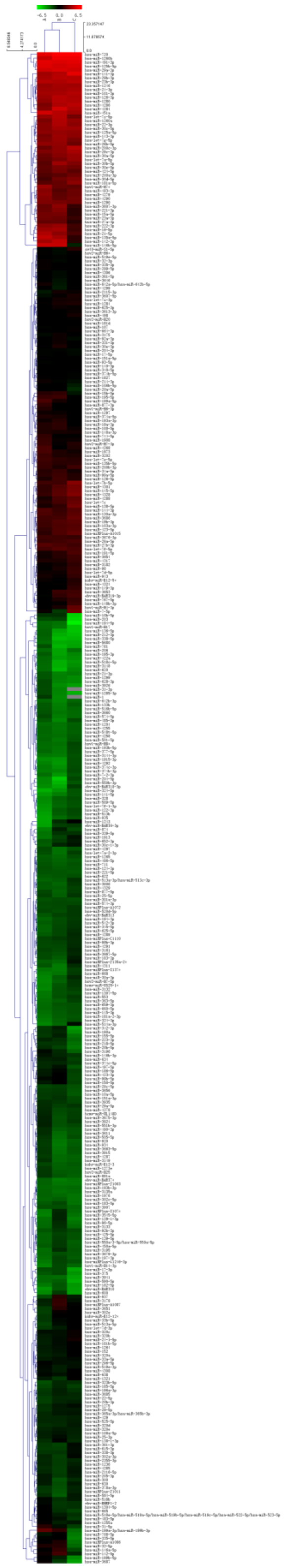

The heat map diagram shows the result of the two-way hierarchical clustering of miRNAs and samples. Each row represents a miRNA and each column represents a sample. The miRNA clustering tree is shown on the left, and the sample clustering tree appears at the top. The color scale shown at the top illustrates the relative expression level of a miRNA in the certain slide: Red color represents a high relative expression level; green color represents a low relative expression levels.

Figure S2. Results of the qRT-PCR.

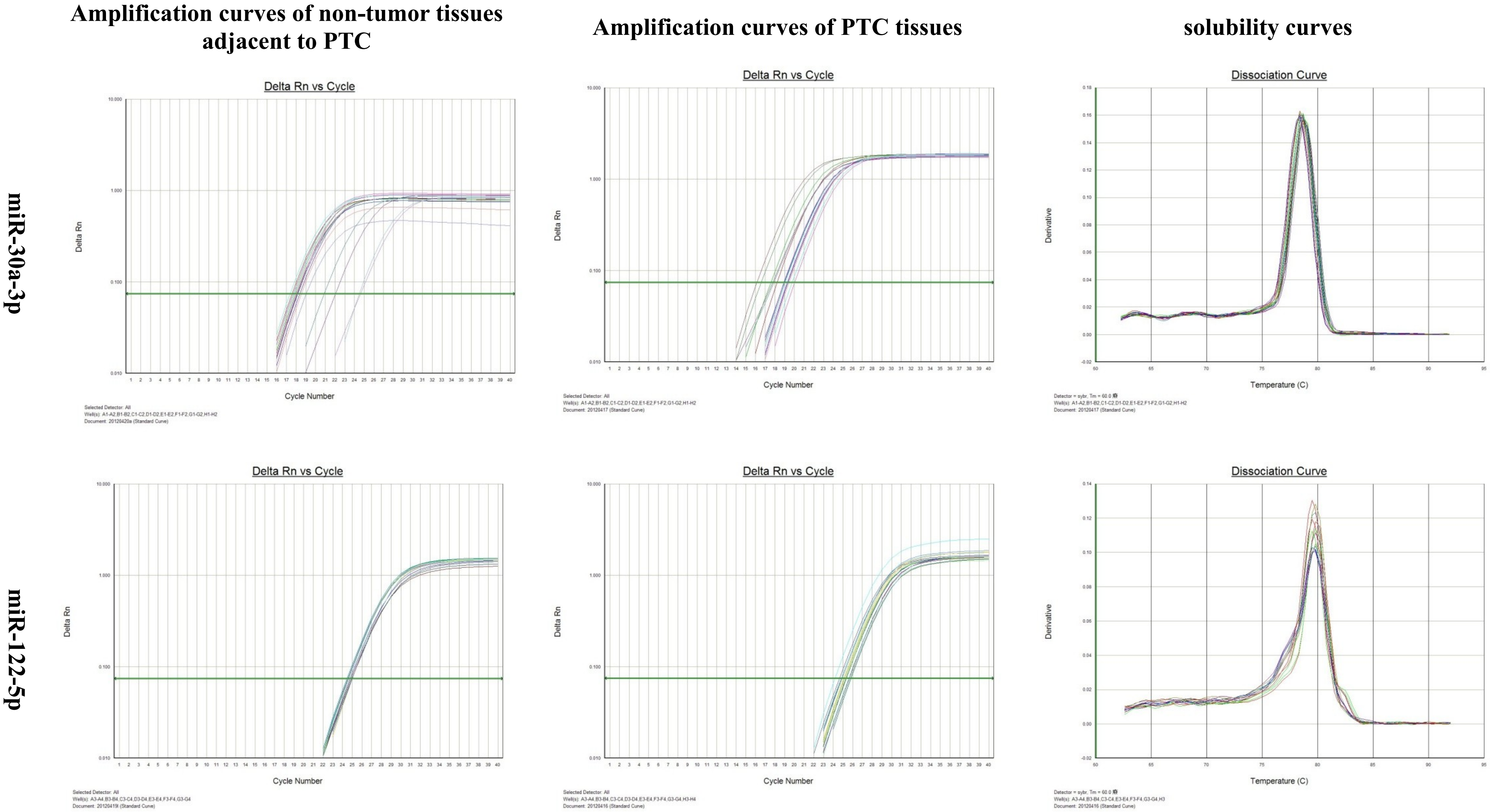

Figure S2. *Cont.*

miR-136-5p

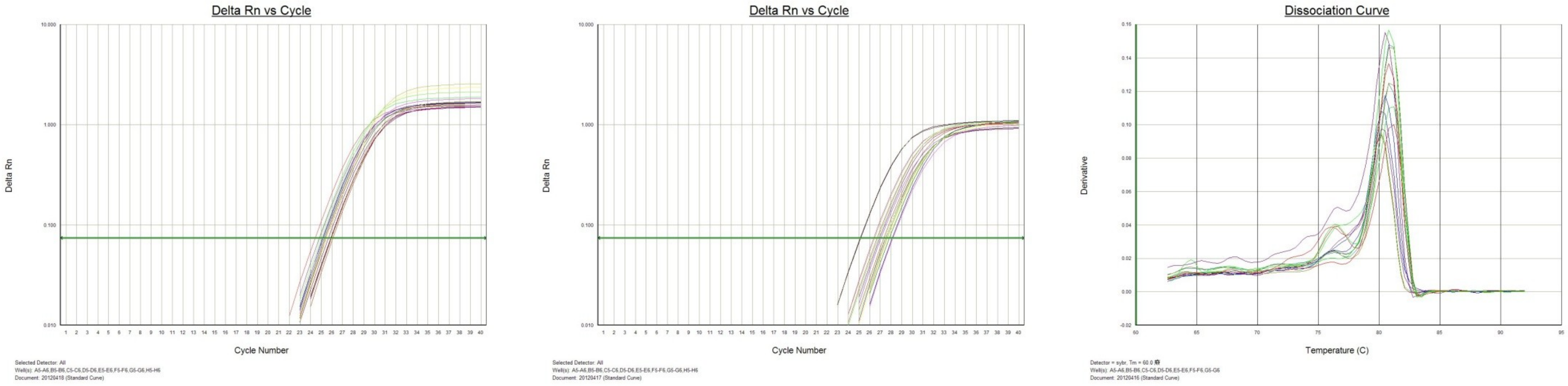

miR-146b-5p

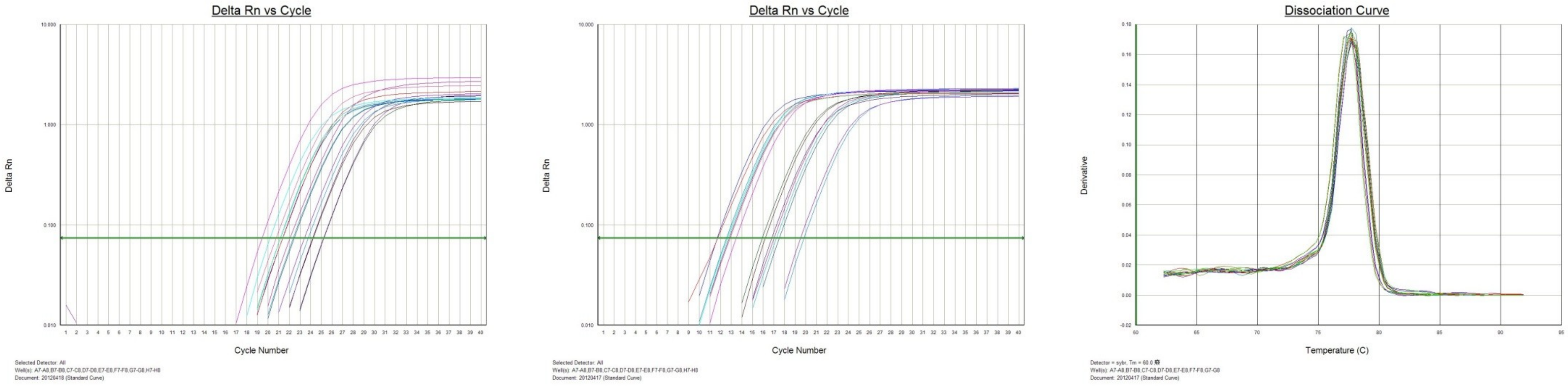

Figure S2. *Cont.*

miR-199b-5p

U6

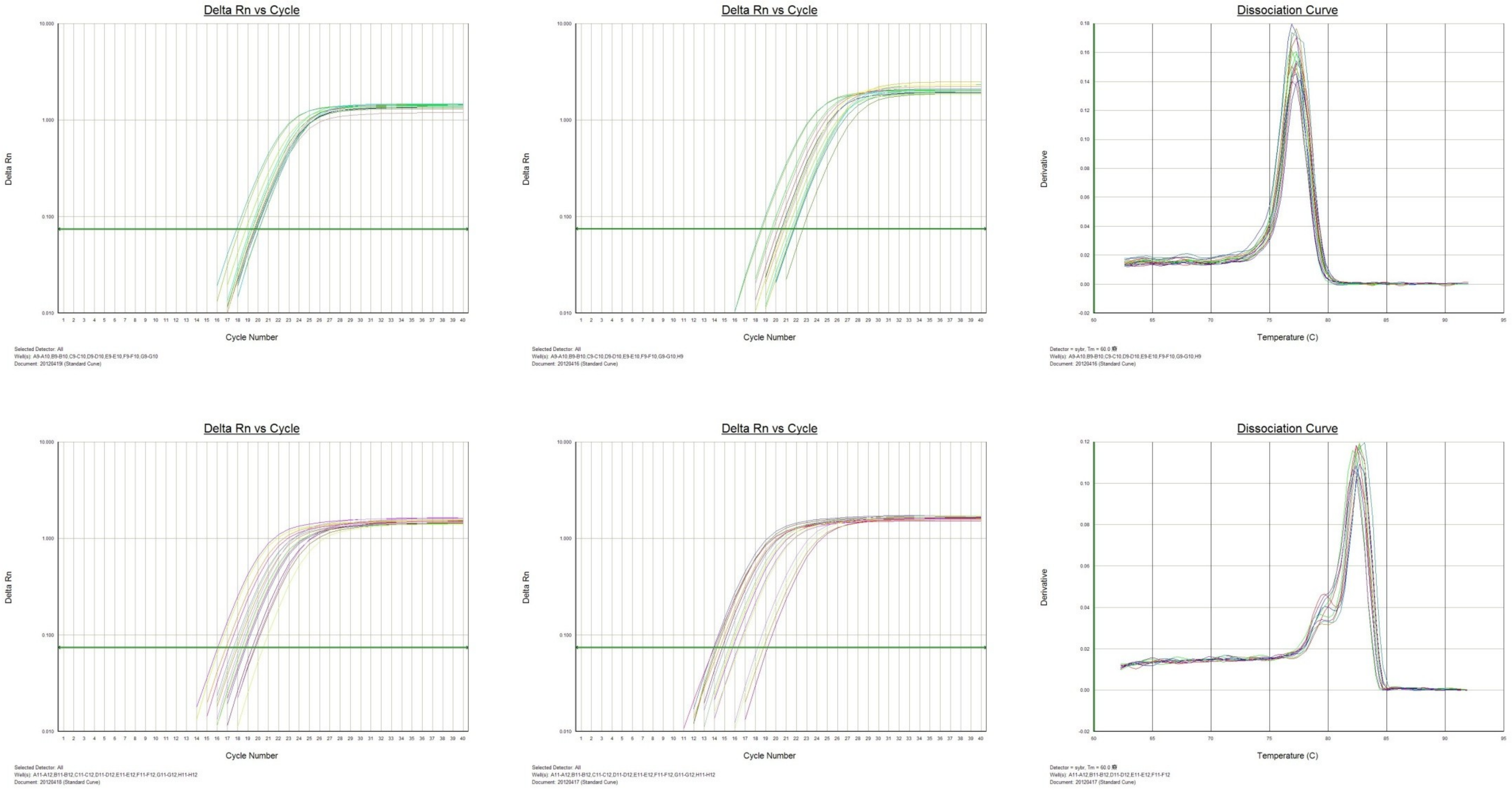

The amplification and solubility curves showed that miRNAs and U6 were all specifically amplified. The amplification curves were smooth, indicating that the samples were completely amplified. The solubility curves were single peaks, indicating specific amplification. The amplification curve for the empty control tube was mainly a horizontal line.
